# Exploring the Housing and Household Energy Pathways to Stress: A Mixed Methods Study

**DOI:** 10.3390/ijerph13090916

**Published:** 2016-09-14

**Authors:** Diana Hernández, Douglas Phillips, Eva Laura Siegel

**Affiliations:** Mailman School of Public Health, Columbia University, New York, NY 10032, USA; doug238@gmail.com (D.P.); els2205@cumc.columbia.edu (E.L.S.)

**Keywords:** stress, low-income housing, energy insecurity, fuel poverty

## Abstract

Chronic stress, known to contribute to negative physical and mental health outcomes, is closely associated with broader issues of material hardship, poor neighborhood conditions, residential instability, and inadequate housing conditions. However, few studies have comprehensively explored pathways to stress in a low-income housing environment. A mixed-methods pilot study investigated the concept of energy insecurity by looking at the impacts of weatherization and energy efficiency interventions on low-income households in the South Bronx neighborhood of New York City. In-depth interviews were conducted with 20 low-income heads of household; participants also completed health, housing and budget assessments. Physical deficiencies, economic hardship, and health issues all interacted to directly and indirectly produce living conditions that contribute to chronic stress. Households with higher stress reported more health problems. Poor quality housing led to coping responses that increased expenses, which in turn increased stress around housing and energy affordability. This study provides further support for the connections between both health and the built environment and between low socio-economic status populations and net negative health outcomes. Energy insecurity is an important contributor to chronic stress in low-income households, and isolating pathways to stress where there is potential for interventions is important for future policy and housing-based strategies.

## 1. Introduction

Living in pervasive poverty is closely linked to chronic stress for low-income populations [1,2,3]. On average, low-income populations suffer from poorer physical and mental health [4], and chronic stress has been identified as a mediating factor between low socio-economic status (SES) and negative health outcomes [5]. The effects of chronic stress on physical and mental health have been mechanistically elucidated through the concept of allostatic load, defined as “the cost of chronic exposure to fluctuating or heightened neural or neuroendocrine response resulting from repeated or chronic environmental challenge that an individual reacts to as being particularly stressful” [6]. This chronic exposure to hormonal stress response causes the body to be more susceptible to adverse health events [7]. According to the National Institutes of Health, chronic stress among adults can lead to more frequent viral infections, high blood pressure, depression, anxiety disorder, and other illnesses [8].

Furthermore, children living under conditions of prolonged adversity and chronic poverty are at greater risk of exposure to hormonal stress response and of experiencing toxic stress, manifested as disruptions of the brain’s architecture and other systems during sensitive developmental periods [9,10]. These disruptions lead to poor stress-management systems, understood as the body’s response to stressors including the secretion of stress hormones, increases in blood pressure, and redirection of blood perfusion to the brain, which develop lower thresholds for responsiveness [11]. This can increase the risk for cognitive impairment and stress-related diseases including cardiovascular disease, asthma, and depression [12]. Research conducted by Evans and others has identified both physical as well as social and environmental exposures as contributors to an elevated cumulative risk exposure in low SES children, and argues that it is the accumulation of these exposures that leads to a state of chronic stress, which in turn leads to low achievement [2]. Due to the wide range and severity of negative outcomes it contributes to in low-income adults and children, it is necessary to understand the sources of chronic stress in these populations.

In low-income populations, poor housing quality, residential instability, lack of affordability, and deprived neighborhood conditions represent significant sources of stress [13,14,15,16]. Within the home, low-income households face myriad challenges associated with housing costs, quality and stability, along with competing expenses and related trade-offs accounting for the numerous possibilities for increased stress and poor mental health. For instance, low-income householders (both private homeowners and apartment renters) often contend with high housing costs relative to low incomes. The strain of unaffordable housing has been associated with hypertension and poor self-rated health [17], and those in the more extreme circumstance of undergoing mortgage foreclosure have been found to have high levels of major depression [18].

Many low-income householders also struggle with energy insecurity. “an inability to adequately meet household energy needs” [19]. This can be driven by economic insecurity in conjunction with inefficiencies that result from poor housing conditions and outdated appliances, lack of maintenance and rising fuel costs [20]. To a certain extent, previous research has examined stress as it relates to housing conditions and energy expenses [21,22]. A 2012 study found that both difficulty with paying fuel bills and poor thermal comfort were independently associated with higher levels of stress [23]. Additionally, a 2004 qualitative study found that fuel poverty could potentially damage mental health “as a result of stress arising from financial worry” [24]. However, these two studies were limited by either using a single variable [23] or no standardized tool to measure stress [24]. Therefore, the relationships between housing conditions, energy insecurity and stress remain poorly understood.

Using both qualitative and quantitative data, this article seeks to advance understanding of the many challenges facing low-income households by identifying the driving forces that may lead to chronic stress in the realms of housing and household energy. Previous research based on the present study examined the impacts of energy efficiency upgrades and showed that improvements in thermal comfort, enhanced health and safety and lower energy expenses were achieved but larger housing issues persisted in spite of benefits [25]. In the current analysis, we examine associations between housing and energy-related issues and stress among participants using the Perceived Stress Scale (PSS), thematic analysis of in-depth interviews, and other survey data. We describe the various pathways to stress among low-income householders that may contribute to net negative health outcomes observed in low SES populations.

## 2. Materials and Methods

The data for this paper are drawn from a mixed-methods pilot study conducted in collaboration with the Association for Energy Affordability (AEA), a community-based weatherization provider. The study investigated the concept of energy insecurity by looking at the impacts of weatherization and energy efficiency interventions on low-income households in the South Bronx neighborhood of New York City [25]. AEA staff identified homeowners and buildings that had recently undergone energy efficiency upgrades and were trained to screen potentially eligible participants for study recruitment. Inclusion criteria for the pilot study were: (1) own or rent a home in the South Bronx; (2) household income between 50 to 150 percent of the federal poverty level (100% is $23,050 for a family of 4 in 2012) following eligibility guidelines for weatherization subsidies; and (3) a 12-month consecutive residence in current housing with intentions to stay at least 6 months after the baseline assessment, so that follow-up interviews could be conducted. In the sample, 55% of households included at least one child under the age of 18, and 40% of households reported less than $20,000 in annual income. This is far below average in New York City, where in 2010–2014 median household income was $52,737 (Uscensus) and average income was $79,950. National household income figures are similar, with a median household income in 2014 of $53,657 and an average household income of $75,738 [26]. This is, however, representative of the South Bronx, where the median income is $23,073 [27]. On the other hand, renters and owners are evenly divided in the study sample, in contrast to New York City, where overall homeownership rates were only slightly over 30% in 2014 (NYU Furman Center). Additional details on participant demographics are presented in Table 1.

### 2.1. Data Collection

Qualitative interviews were conducted with the head of household to explore experiences with energy insecurity and housing conditions, and their connection to health and economic problems. If there was more than one adult in the home, the reference person for AEA paperwork was considered the head of household. Participants also completed several assessments at baseline, including (a) a health survey to measure household health status and healthcare utilization patterns, the Perceived Stress Scale; (b) a retrospective utility audit to understand energy consumption and expenses; and (c) a budget audit that itemized household expenses including housing, utility, food, transportation, personal, child and medical care, telecommunications, pet, and entertainment expenses.

### 2.2. Measures

Participants were asked a series of questions pertaining to housing, energy, household economics, health and stress. Questions were derived from validated surveys and scales as described below [28,29,30,31,32,33]:

Housing: the US Census Bureau American Housing Survey (AHS), including questions on building structure and maintenance, occupancy, and pest infestation.

Energy: the Department of Health and Human Services Home Energy Insecurity Scale Survey (HEIS), the Department of Energy S4 Occupant survey.

Household Economics: the budget audit estimates each participant’s expenses on a monthly basis, by category. Included are expenditures for housing, utilities, food, transportation, children, personal care, medical expenses, entertainment, pets, and miscellaneous. Since food purchased with Supplemental Nutrition Assistance Program (SNAP) benefits could be considered as not an expense by some participants, all analyses involving food expenses were conducted including and excluding food purchased with SNAP benefits. No significant differences were observed between the two.

Health: the asthma survey, based on the NYC Neighborhood Asthma and Allergy Study (NAAS), posed questions related to household members’ asthma and other respiratory conditions as well as disease severity and care management. Questions adapted from the SF-12 health survey [34] covered participants’ physical and mental health and their effects on daily life activities.

Stress: the Perceived Stress Scale (PSS) is the most commonly used measure of perceived stress [35]. It is designed to measure how unpredictable, uncontrollable, and overloaded participants find their lives. Participants answer 10 questions on a scale of 0–4. Answers are summed to determine an individual PSS score, with higher numbers reflecting higher levels of perceived stress. For this work we used the 10-item version of the PSS (with a possible score range of 0–40) as it has been tested for reliability and validity more frequently than the 14 and 4 item versions [28,35].

### 2.3. Data Analysis

The present analysis explored associations between stress, housing conditions, household expenses, and health that emerged from the quantitative measures and qualitative themes. Quantitative analysis was conducted using SAS to examine associations between higher levels of stress and other survey variables using linear regression and the Wilcoxon Mann-Whitney U-test in the case of non-parametric data. Sensitivity analyses imputing missing data points by various methods determined that models were robust, and statistics given are for the data as originally reported. The research team then conducted a thematic analysis based on qualitative data to further explore issues pertaining to increased stress levels, and to elucidate the associations observed in the quantitative analysis.

## 3. Results

**Figure 1:**
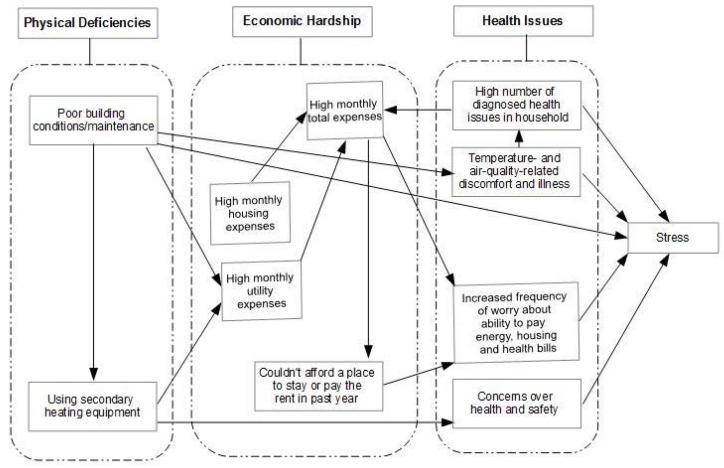
Housing, energy and health pathways to stress.

*Perceived Stress.* The mean PSS score among participants was 12.5 (SD = 8.61), with the highest score being 27 and the lowest being 0 (the possible range is 0–40, with 0 representing no perceived stress). This mean score falls within the range reported in several national surveys that utilized the PSS [36]. In our sample, the PSS analysis identified several variables that were associated with higher perceived stress. Triangulating this quantitative data with themes revealed in the qualitative analysis, we observed three main pathways to stress including: (1) Physical deficiencies (pertaining to quality of building conditions and heating systems); (2) Economic hardship (housing and utility expenses); and (3) Health issues (physical and mental health). As depicted in Figure 1, some variables were more directly associated with stress while others represented associations between one or more factors leading to stress.

### 3.1. Physical Deficiencies in the Home Environment

Physical deficiencies contributed directly and indirectly to stress. Lack of thermal comfort caused by poor building conditions was a direct source of stress for participants, who reported being exposed to uncomfortably cold temperatures on a near-daily basis.

“My dogs couldn’t sleep, they would shiver... I mean literally, I had to put clothes in there, I gave them the heater, I put the heater for my dogs. And my wife was sleeping in a sweater like this, hoodied up, sweatpants, socks... she can’t stand it and it really affects her. And, it was a rough winter here.”

*—*10011

“The front room, that’s why this is out here (pointing to bed in living room) because it’s like I’m not sleeping in there... F***ing cold, I mean it’s freezing. In there, I mean you can probably see your breath in the air. That’s how cold it is in that room alone.”

*—*10010

Participants cited problems with poor insulation, damaged or improperly sealed doors and windows, and inadequate heating systems as the main factors impacting their thermal comfort and inadequacies in household energy more broadly. For private homeowners, the physical deficiencies were generally a result of not being able to independently afford home improvements and energy efficiency upgrades, while for renters the issues were a result of improper maintenance and upkeep by landlords.

Indirectly, these physical deficiencies also led to additional stress associated with problems of home safety and security as noted by homeowners and renters alike:

“I would get snow and water (inside my home from) the draft on the door... If the wind would blow one way, we would have snow piling up in the house. I couldn’t get that fixed. I had the front door broken with no lock that you could just push it with a finger for years, because I couldn’t afford a new door... So for a few years I had no security whatsoever for the door.”

*—*10003

“When I moved in here, the window in the kitchen, at the fire escape wouldn’t stay open. So you would have to prop it up... that’s messy, because it’s the window. It’s a method of escape and if you can’t keep the window open or to get out, it’s a dead weight window. And that took three months. And that was only because the city came and did that...”

*—*10010

Unsurprisingly, living in poorly insulated homes was significantly associated with the use of secondary heating equipment (parameter estimate: 0.09 (95% CI: 0.01–0.70), *p* = 0.02); 50% of participants in the sample reported having used secondary heating equipment to compensate for inadequate thermal conditions. In turn, the use of secondary heating equipment was associated with a significantly higher level of perceived stress (parameter estimate: 0.86 (95% CI: 0.75–0.99), *p* = 0.03). In association with the use of secondary heating equipment, participants expressed concerns related to physical health and increased energy costs, both of which led to increased stress.

Interviewer: “Did you ever use your stove or oven to provide heat?”

Respondent: “Yes... I only did it one time cause like I was telling her, running that gas is not healthy at all, but it was unbearable so it had to be done.”

—10011

Secondary heating equipment used to keep homes at an adequate temperature can be potentially dangerous, and includes the use of space heaters, ovens and stoves. Use of these heating sources contributes to harmful exposures and increases the risk of fire and fire-related injury and death [37,38,39,40]. According to the National Fire Protection Association, space heaters are responsible for a third of all home heating fires, and 81% of all home heating fire deaths [37,38]. Oven or stove use for heat also increases the risk of carbon monoxide poisoning and nitrogen dioxide exposure [39,40].

The use of secondary heating equipment also raises utility bills. A non-parametric test showed that the use of secondary heating equipment was associated with increased monthly utility expenses (*p* = 0.006). Participants who used secondary heating equipment had significantly higher average monthly utility bills ($418.60 ± $161.99) compared to those who did not ($200.56 ± $129.99).

“Right now the (utility bill) I just got was not that high, it was two sixty five for one month. And then next month it’s gonna be a little higher, could be more... because my mother uses the electric heater.”

—10009

The physical deficiencies discussed here had direct links to stress through chronic exposure to cold temperatures in the home. Indirect links included concerns over health and safety of participants and their families, and the need to use secondary heating equipment leading to further concerns about safety, and about higher energy expenses (Figure 1).

### 3.2. Economic Hardship

Economic hardship were a second major source of stress within the study population. The “trifecta of insecurity”, as previously described by Hernández [20] is observed in the housing, food and energy costs reported as the three largest expenditures within the monthly budget. Increases in total monthly expenses were significantly associated with increases in levels of perceived stress (parameter estimate: 0.005 (95% CI: 0.002–0.008), *p* = 0.002). A budget audit, detailed in Table 2, shows category-specific expenses as well as the sum total of average monthly expenses across the sample, stratified by home ownership status.

As confirmed in Table 2, the budget portion of the in-depth interviews indicated that housing and utility expenses were the highest expenses for most participating households. Housing, in particular, represented the largest expense for participants, with owners paying more for both housing expenses and utilities than renters. Several participants discussed how their high monthly expenses resulted in frequent worry about their ability to pay household bills.

“I was trying not to default on the mortgage. That was the main thing. The water bill was here, that was there, and you know, it was rough... you know, the anxiety would build. When it comes, the bills show up, and then there’s no money, and school started, and the kids have this, and then, you know, of course, it puts you in an emotional state.”

*—*10002

“I’ve gotten a light bill for $1,100 dollars. That’s impossible, I don’t own a store. That is impossible. And who has to suffer and pay it? We did... How you gonna get that when you gotta pay $1,300 dollars rent? You know, we all have budgets, so. Yes it has been rough, as far as when it comes to that light.”

*—*10011

“When I do the level billing at the end of the year, there’s always a large amount that I have to pay. So then I always have to tell them, you know, I can’t pay it all. It’s always in the thousands. So even though the level billing is great, because I’m fixed on one thing, but at the end of the year that’s when the stress level comes.”

*—*10003

Not only did many participants struggle to pay their monthly rent or mortgage, but also high monthly energy expenses were a critical source of concern. Increases in the frequency of participants worrying about not being able to pay their home energy bills were strongly correlated with increases in perceived stress (parameter estimate: 6.09 (95% CI: 4.43–7.74), *p* < 0.001), confirmed by participant interviews. As demonstrated in the results related to physical deficiencies, poor housing conditions often exacerbated high monthly energy expenses.

### 3.3. Health Issues

During the in-depth interviews, participants discussed how their health issues, both physical and mental, were sources of stress, particularly among those with a high number of diagnosed ailments, as indicated by a statistically significant association between the number of health problems affecting household members and perceived stress levels (parameter estimate: 2.25 (95% CI: 0.39–4.1), *p* = 0.02).

“I have low blood pressure, so with the asthma it’s a problem. Last year one Sunday I was sweeping the leaves. The next day I couldn’t even breathe, I had to go to the doctor. I ended up in the hospital period... So every time I get sick from the asthma it’s a problem, because of the medication issues. I can’t take inhalers.”

*—*10009

“When we didn’t have a furnace... they would bundle up. Then her boyfriend loaned us three heaters... you had to do what you had to do. You know?... We had to deal with a lot of colds, my asthma.”

—10003

“He’s gone through depression because he couldn’t find a job and he felt horrible. And a couple days ago he says, ‘Mom, I feel bad going to school... I’m a man now’... But, so he’s stressed out. And he’s been depressed with this now.”

—10003

At the same time, the stress associated with poor health status was not only a result of the health issues themselves, but also from the financial strain associated with the healthcare costs.

“I was hospitalized in February and so they made a single payment of like 9000 dollars and so there’s an outstanding balance of like sixty something thousand dollars and so sometimes they call me... how am I going to pay? Imagine that quantity.”

—10008

Interviewer: “Now tell me a little about the anxiety you were describing, like feeling anxious…”

Respondent: “Yeah, that’s a lot. Because I don’t know, like when I got injured it was like what’s gonna happen? And then I had the misfortune that the insurance company for compensation has been horrible. They delay my payments for three, four months, and I’ve had to fight. And I couldn’t go to work... the anxiety of you know, when was my next check? I was down to my last payment. After that I didn’t know how I was going to pay.”

—10003

Additional health issues associated with stress were referenced in the previous section. For example, as noted above, some participants living in poor quality housing resorted to using their stove or oven to provide heat, which resulted in stress from concerns about toxic effects of gas fumes and risk of fire or contending with the discomfort of extreme home temperatures. Poor quality housing also caused concerns about health and safety from lack of security, leading to increased stress.

## 4. Discussion

The results of the present study indicate that there is no singular factor responsible for stress among participants, but that a variety of physical deficiencies, economic hardship, and health issues convene to create conditions that can result in chronic stress. Of the three main themes described here, physical deficiencies both contributed directly to stress as well as indirectly to economic and health issues that lead to stress, demonstrating the strong links between housing conditions and stress along direct and indirect routes. Poor housing conditions led to coping strategies (i.e., use of space heaters, stove/oven use for heat) that increased energy expenses and also increased participant’s concerns over health and safety. These factors, in turn, contributed to stress independently and through associations between factors. As such, this article offers a novel assessment of the intersecting links between housing conditions, energy insecurity, economic hardship, health and stress. As exposure to chronic stress leads to increased susceptibility to adverse health events [7], which in turn leads to increased stress, a self-reinforcing cycle develops. These results help to identify areas where interventions can be deployed to help mitigate the impact of the upstream determinants of stress.

Overall the results point to a “trifecta of insecurity”, where physical, economic, and health issues all contribute to stress among our study participants [20]. Physical deficiencies within the built environment and poor housing conditions were directly associated with stressand also led to uncomfortable living conditions and coping mechanisms that increased concerns about the health and safety of household members. These concerns on health and safety in turn were also associated with increased stress. Similarly, poor building conditions led to the use of secondary heating equipment that significantly increased utility expenses, contributing to the economic issue of significant financial burden.

As was seen in Table 1, 35% of the participating households were inhabited by seniors. This population is particularly vulnerable to health conditions such as arthritis, respiratory illness, cardiovascular conditions and other chronic health conditions that can be affected by their housing conditions. Given that another 55% of participating households were inhabited by children (Table 1), who are also more vulnerable to asthma and other respiratory illnesses, this population can be considered highly vulnerable to health effects from poor housing conditions.

Frequent worry about being able to pay energy and household bills, concerns over the health and safety of household members, and high numbers of health issues in the household were all sources of stress among study participants. For households with high numbers of health issues, stress was a result of the health issues themselves, as well as worries over the financial costs associated with treatment and care. Participants who were consistently worried about the health and safety of their families, about their ability to pay their bills, and who were also suffering from health problems, were likely to report increased stress.

Given the known associations between chronic stress, allostatic load, and negative health outcomes, it is unsurprising that households with higher stress would report more health problems. As stress increases susceptibility to adverse health events [7], it is more likely that this relationship is cyclical as opposed to one of reverse causation, although it should be noted that the present cross-sectional study by design does not examine temporality. Furthermore, this research identifies energy insecurity as an important contributor to chronic stress in low-income households, isolating these pathways to stress where there is potential for interventions.

Previous research has demonstrated that improvements to housing conditions targeting thermal comfort and energy efficiency can improve both the physical and mental health of household members [41,42,43,44,45]. There are several opportunities to address poor housing quality through policy. Programs providing no-cost or low-cost funding or incentives for apartment and home improvements, such as the Weatherization Assistance Program, can address many of the structural and energy efficiency problems that lead to uncomfortable living conditions, higher utility expenses, and stress. However, funding for the Weatherization Assistance Program has been historically low in recent years. Educating the public through awareness campaigns to promote safe, efficient and affordable thermal comfort solutions could aid these communities in making informed decisions.

Another avenue to address stress and health disparities is through housing-based interventions. Property owners investing in their buildings through physical repairs and energy efficiency upgrades can experience a variety of benefits. Investment in building improvements can increase property values and reduce energy expenses [25]. Furthermore, such improvements can increase the comfort and well-being of building residents and improve tenant-landlord relationships [25].

An additional pathway for intervention in housing conditions is through the Department of Housing and Urban Development (HUD). In the past, HUD has worked with researchers such as Dr. James Krieger on the Healthy Homes Program, aimed at removing lead and other potential hazards from the home [46,47]. This work has set a precedent for addressing housing problems linked to health, and energy efficiency interventions may be the next target area. A 2014 study based in Boston found lower environmental exposures to PM_2.5_, NO_2_, and nicotine in green homes as compared to control homes [48], and other energy efficiency studies are currently underway funded by HUD and the Centers for Disease Control and Prevention.

### Limitations

The small sample size and specific location of this pilot study limit the generalizability of results. Moreover, the strength of the quantitative analysis was limited to basic statistical tests rather than more sophisticated methods including multivariable analysis given the limited sample size. In the case of the budget audits, participants’ self-reported expenses; expense data was not validated through receipts or bank statements and in some cases reporting was incomplete or unclear. Accordingly, the qualitative interviews were an integral part of the data analysis and results, as they helped complement the quantitative results, as well as uncover issues that were not evidenced in the quantitative portion of the study. Despite these limitations, the findings were supported by quantitative results, qualitative results, or both, and merit more attention in future studies.

## 5. Conclusions

This study helps illuminate the links between low-income housing conditions, energy insecurity, and stress. It also provides further support for the connections between health and the built environment and between low SES populations and net negative health outcomes. The aforementioned policies and housing based interventions are viable options for improving housing conditions and reducing stress among low-income households. However, more needs to be done to examine health and socioeconomic status in the housing environment in order to design the most effective interventions.

## Figures and Tables

**Table 1:** Demographics.

	n (%)
Gender	
Male Female	6 (30) 14 (70)
Race/Ethnicity	
Non-Hispanic Black or African-American Hispanic or Latino	4 (20) 16 (80)
Primary Language Spoken	
English Spanish	10 (50) 10 (50)
Household Composition	
Households with Children (<18 y/o) Households with Elderly (>60 y/o) Households with Adults only (18–59 y/o)	11 (55) 7 (35) 2 (10)
Age of Survey Participant	
18–30 31–45 46–60 60+	2 (10) 3 (15) 8 (40) 7 (35)
Annual Household Income	
<$10,000 $10,000–$19,999 $20,000–$29,999 $30,000–$39,999 $40,000–$49,999 >$50,000	3 (15) 5 (25) 2 (10) 2 (10) 2 (10) 6 (30)
Housing—Rent/Own	
Private Homeowner Rental Apartment	10 (50) 10 (50)
Health Problems	
0–1 2–3 4+	5 (25) 9 (45) 6 (30)

**Table 2:** Budget audit.

Budget Category	Description	Avg. Homeowner	Avg. Renter
Housing	Rent or Mortgage, Insurance, Taxes, etc.	$1,310	$1,158
Food	Groceries and Eating Out	$389	$522
Utilities	Electricity and Gas, also Heating Fuel and Water for Homeowners	$393	$245
Telecommunications/Home *	Phone/Cable/Internet	$308	$246
Transportation	Public Transportation Costs, Taxis, Automobile Fuel, Automobile Insurance, etc.	$286	$179
Children	Clothing, School Tuition, Extracurricular Activites, etc.	$133	$119
Personal Care	Clothing, Haircuts, Manicures/Pedicures, etc.	$83	$84
Out of Home Entertainment	Movies, Theater, Music, Sporting Events, etc.	$13	$132
Medical Expenses *	Medical Care and Prescriptions	$53	$16
Other	Other Expenses, including Charitable Donations, Religious Tithes, Remittances, etc.	$39	$15
Pets	Food, Medicine, etc.	$3	$20
Total Monthly Expenses		$2,902	$2,736

* Due to incomplete data for indicated variables, average expenses separated by budget category may not add up to average total expenses.
